# Spatial patterns in host-associated and free-living bacterial communities across six temperate estuaries

**DOI:** 10.1093/femsec/fiad061

**Published:** 2023-06-02

**Authors:** Alessandra L Suzzi, Michael Stat, Troy F Gaston, Megan J Huggett

**Affiliations:** School of Environmental and Life Sciences, The University of Newcastle, Ourimbah, NSW 2258, Australia; School of Environmental and Life Sciences, The University of Newcastle, Ourimbah, NSW 2258, Australia; School of Environmental and Life Sciences, The University of Newcastle, Ourimbah, NSW 2258, Australia; School of Environmental and Life Sciences, The University of Newcastle, Ourimbah, NSW 2258, Australia; Centre for Marine Ecosystems Research, School of Science, Edith Cowan University, Joondalup, WA 6027, Australia

**Keywords:** bacterial communities, estuary, free-living, host-associated, microbiome

## Abstract

A major goal of microbial ecology is to establish the importance of spatial and environmental factors in driving community variation. Their relative importance likely varies across spatial scales, but focus has primarily been on free-living communities within well-connected aquatic environments rather than less connected island-like habitats such as estuaries, and key host-associated communities within these systems. Here we sampled both free-living (seawater and sediment) and host-associated (estuarine fish hindgut microbiome, *Pelates sexlineatus*) communities across six temperate Australian estuaries spanning ∼500 km. We find that spatial and environmental factors have different influences on these communities, with seawater demonstrating strong distance-decay relationships (*R* = −0.69) and significant associations with a range of environmental variables. Distance-decay relationships were weak for sediment communities but became stronger over smaller spatial scales (within estuaries, *R* = −0.5), potentially reflecting environmental filtering across biogeochemical gradients or stochastic processes within estuary sediments. Finally, *P. sexlineatus* hindgut microbiome communities displayed weak distance-decay relationships (*R* = −0.36), and limited variation explained by environmental variables, indicating the significance of host-related factors in driving community variation. Our findings provide important ecological insights into the spatial distributions and driving forces of both free-living and host-associated bacterial patterns across temperate estuarine systems.

## Teaser

Environmental and spatial factors have greater influence on estuarine free-living bacterial communities in comparison to host-associated communities across six temperate estuaries spanning 500km.

## Introduction

Microorganisms play crucial roles in global biogeochemical cycles, particularly critical to the functioning of dynamic environments such as estuaries (Hanson et al. 2012, Zhang et al. 2022). A key focus of marine microbial ecology is the study of spatial patterns and related environmental factors in order to understand the mechanisms responsible for the generation and maintenance of diversity (Van der Gucht et al. 2007, Hanson et al. 2012). The contributions of spatial factors and local environmental conditions are considered to be major competitive forces in driving microbial biogeography; however, these are thought to be largely scale-dependent: on a global scale, spatial separation tends to overwhelm local environmental effects; at small scales, environmental effects are the major determinants; and at intermediate scales, both local environmental conditions and spatial factors are important drivers of community variation (Martiny et al. 2006, 2011).

The Baas-Becking hypothesis “everything is everywhere, but the environment selects” (Baas-Becking 1934) implies that all microorganisms are globally distributed, with local environmental conditions driving selection of distinctive assemblages (De Wit and Bouvier 2006). This early hypothesis has been heavily debated by numerous studies, which have demonstrated geographic separation independent of changes in the environment, indicating dispersal limitation (Langenheder and Ragnarsson 2007). One of the most commonly studied biogeographic patterns is the distance-decay relationship, which refers to decreasing community similarity with increasing geographic distance (Hanson et al. 2012, Zhang et al. 2022). Similar to patterns observed for macroorganisms, distance-decay relationships are thought to be stronger in heterogeneous habitats and for island-like habitats compared to continuous environments (Prosser et al. 2007, Zinger et al. 2014); however, patterns are influenced by both deterministic and stochastic processes (Wang et al. 2013), highlighting the importance of identifying major assembly processes governing community diversity.

In order to increase our understanding of the contributions of spatial and local environmental effects on microbial communities, it is also crucial to consider different bacterial habitats (e.g. free-living vs host-associated) (Taylor et al. 2005). While distributions of free-living microorganisms may be strongly driven by dispersal limitation and environmental filtering (Fuhrman et al. 2008, Wietz et al. 2010, Liu et al. 2018, Zhang et al. 2022), host organisms provide unique environmental conditions that differ from those in the surrounding seawater or sediments and may therefore function as island habitats, allowing for allopatric speciation of symbiotic microbes living in physically separated hosts, resulting in distinct host-associated communities and contributing to distance-decay patterns (Papke and Ward , Taylor et al. 2005). Dispersal of host-associated communities is therefore likely dependent on the movement or migration of the host, with these communities comparatively more buffered from the effects of environmental conditions (Dickey et al. 2021). Despite this, the biogeography of host genotypes and local environmental-genotype interactions have been documented, along with environmental filtering through diet in shaping associated microbiomes (Spor et al. 2011, Wagner et al. 2016, Loo et al. 2019, Baltrus 2020, Baldassarre et al. 2022). Evidence indicates that both host-related and environmental factors drive variation in microbial communities associated with key marine hosts such as sponges and corals (Taylor et al. 2005, Luter et al. 2015, Rubio-Portillo et al. 2018, Easson et al. 2020); however, knowledge of the relative importance of these remains limited, especially in important estuarine host organisms such as fish.

Within coastal systems, fish have great ecological, economic, and cultural significance (Schlacher et al. 2005), representing an important study organism for addressing questions for host-microbe associations and microbial diversity. Despite their significance and the recognition that fish gut microbiome interactions are important for host fitness, metabolism, and immunity, host-microbiome interactions in fish are understudied in comparison to other vertebrates (Ghanbari et al. 2015, Colston and Jackson 2016). Fish are constantly exposed to the surrounding seawater and sediments via dietary uptake; however, there is evidence that gut microbial communities are distinct from those in the surrounding environment (Navarrete et al. 2012, Li et al. 2015), suggesting water-borne dispersal between hosts may be negligible. While such spatial patterns have been established in free-living microbial communities (Zinger et al. 2014, Wang et al. 2015, Liu et al. 2018, Zhang et al. 2022), investigations into these patterns have been largely overlooked for hosts occupying dynamic and complex estuarine systems such as fish.

Both environmental and spatial variability are important drivers of microbial variation in continuous aquatic environments, but these patterns are less clear in less connected, island-like habitats (Hanson et al. 2012), such as estuaries. Estuaries represent unique hotspots of biogeochemical cycles with high microbial biodiversity supported along biogeochemical gradients (Webster et al. 2015, Liu et al. 2018, Zhang et al. 2022). Here, we sampled both free-living and host-associated microbial communities across an intermediate scale of ∼500 km, spanning six east Australian estuaries, and aimed to address the following questions: what influence do spatial and environmental factors have on microbial communities, and does this differ for free-living vs host-associated communities? We hypothesized that: (a) distance-decay relationships would be stronger for free-living than host-associated communities due to limited movement of environmental bacteria between estuaries; (b) at the intermediate scale sampled in this study, spatial effects and local environmental variation would both contribute to spatial patterns across estuaries; and (c) free-living communities would be more strongly shaped by local environmental variation than host-associated communities due to their closer association with the environment.

## Methods

### Field

**Figure 1.**
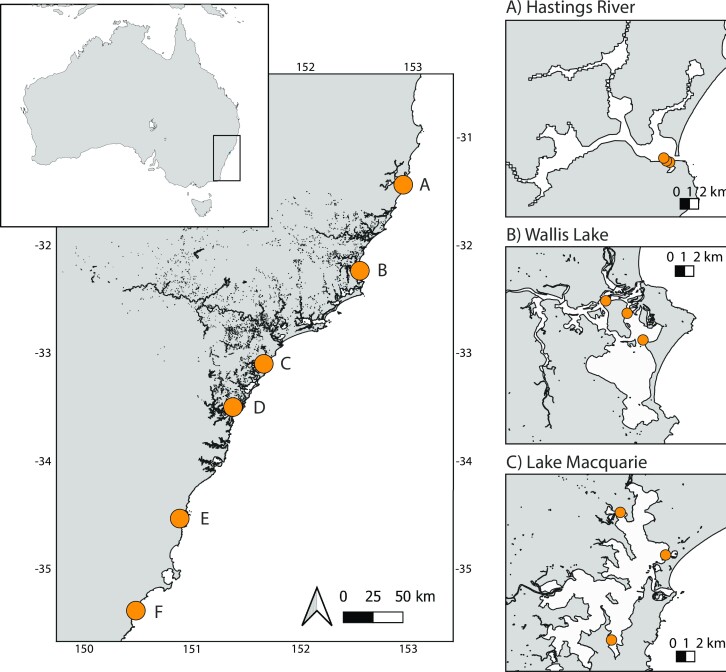
Map of six east Australian estuaries sampled and sampling locations within each estuary: (A) Hastings River, (B) Wallis Lake, (C) Lake Macquarie, (D) Brisbane Water, (E) Lake Illawarra, and (F) Burrill Lake.

This research was conducted under the University of Newcastle Animal Ethics Protocol A-2020-026. Six estuaries along the NSW coastline were selected for sampling, based on available seagrass habitat and distribution of the estuarine fish *Pelates sexlineatus* (eastern striped grunter): Hastings River, Wallis Lake, Lake Macquarie, Brisbane Water, Lake Illawarra, and Burrill Lake (Fig. 1). *Pelates sexlineatus* is a common estuarine fish selected for its broad distribution in seagrass meadows along the south-east coast of New South Wales (Pollard 1984, Trnski and Neira 1998, Smith and Suthers 2000). Seawater, sediments, and *P. sexlineatus* individuals were collected from three sites within each estuary between October and November 2020. The following water quality parameters were measured at each site using a Horiba U-50 water quality meter: temperature (precision of 0.01°C), salinity (0.1 ppt), pH (0.01), turbidity (0.1 NTU), and dissolved oxygen (0.01 mg/L). Seawater was collected in sterile bottles (rinsed with 10% bleach solution) for amplicon sequencing (*n* = 5), with ∼800 ml from each sample filtered onto 0.2 μm pore-sized Sterivex filters, depending on the amount of particulate organic matter present. Sediment samples (50–60 ml) were collected using a 50 ml Luer Lock syringe plunged vertically into the sediment to ∼100 mm depth, for sediment granulometry and the determination of organic matter. Surface sediments were also collected for amplicon sequencing (*n* = 5) by scraping the upper 1 cm layer into sterile 15 ml tubes. *Pelates sexlineatus* were collected using a 10-m seine net pulled through seagrass beds for amplicon sequencing of the hindgut microbiome (*n* = 5). All samples were immediately stored on ice and processed within 6 h of collection.

### DNA extraction and 16S rRNA gene sequencing

*Pelates sexlineatus* total length (mm) and weight (g) were recorded, and fish hindgut contents removed (∼0.25 g). A 0.25 g sample from collected sediments were stored at −80°C until DNA extraction. Qiagen DNeasy PowerSoil Kits were used to extract DNA from fish and sediment samples, and Qiagen DNeasy PowerWater Kits were used for seawater samples. Extraction blanks containing no sample were processed with each batch to serve as controls. Sample quantity and quality were checked using a NanoPhotometer NP80. Bacterial communities were characterized using 16S rDNA gene amplicon sequencing. The V1–V3 region of the 16S rDNA gene from prokaryotes was amplified using universal primers 27F (AGAGTTTGATCMTGGCTCAG) and 519R (GWATTACCGCGGCKGCTG) attached with Illumina adaptors in PCR with the following cycling conditions: 95°C for 10 min, then 35 cycles of: 94°C for 30 s, 55°C for 10 s, and 72°C for 45 s, with a final extension of 72°C for 10 min. PCR products were sequenced using the Illumina Miseq v3 (2 × 300 bp) platform at the Ramaciotti Centre for Genomics at the University of New South Wales. Resultant amplicons were processed using the R pipeline DADA2 with default parameters (Callahan et al. 2016), including the removal of potential chimeras. Reads were clustered to produce amplicon sequence variants (ASVs) and aligned to the SILVA v132 database (Yilmaz et al. 2014) for taxonomic assignment. The dataset was further cleaned by removing singletons and those identified as non-bacterial or originating from chloroplasts. Of the 90 samples that were collected for each sample type, 65 fish hindgut samples were successfully sequenced, as well as 75 sediment samples, and 50 seawater samples (Supplementary Table S1). Cleaned data were rarefied to 4276 reads per sample (see Supplementary Figure S1 for rarefaction curves on raw and rarefied data, and Supplementary Table S1 for a summary of samples sequenced and retained after rarefaction). Samples with reads below this threshold (seven fish hindgut samples and two seawater samples) were removed in order to retain diversity after rarefaction.

### Statistical analysis

Statistical analysis was carried out in RStudio using the “vegan” package (R Core Team 2018, Oksanen et al. 2019). Microbial community data was analyzed using the “phyloseq” package (McMurdie and Holmes 2013), with each sample type (seawater, sediment, and fish hindgut) analyzed separately. Diversity metrics were calculated using rarefied sequence data. Linear regression models were employed to test correlations between bacterial community similarity (Bray–Curtis similarity) and geographic distance (km^2^) and environmental variables (Euclidean distances), and distance-decay slopes were visualized using scatterplots. PERMANOVA with pairwise comparisons was used to investigate significant differences in community composition (based on Bray–Curtis dissimilarity) between estuaries, and PERMDISP was further employed to investigate differences in community variance across estuaries using the “betadisper” function (Oksanen et al. 2019). ANOVA with Tukey’s HSD was employed to assess significant differences in alpha diversity (observed richness and Shannon diversity).

The following environmental variables were extracted from the NSW Estuary Health Monitoring, Evaluating, and Reporting (MER) program on the SEED NSW database ([dataset]* Department of Planning and Environment 2020) for each of the sampled estuaries: average depth (m), flushing time (days), catchment area (km^2^), catchment cleared (%), urbanization (%), estuary surface area (km^2^), and estuary volume (ML). NSW estuarine macrophyte data was obtained from the Fisheries NSW Spatial Data Portal ([dataset]* NSW Department of Primary Industries), and seagrass, mangrove, and saltmarsh area (km^2^) for each estuary was calculated in QGIS 3 (QGIS.org 2022 et al. 2022) using the GroupStats plugin (HenrikSpa 2021). Environmental variables used in the study are presented in Supplementary Table S2 and were shown to change significantly over the sampling area (with geographic distance) (see Supplementary Table S3). Environmental variables were checked for collinearity, and those that were highly correlated with other variables (*R* ≥ 0.8) were removed from further statistical analyses. Mantel and partial mantel tests were used to test the correlation between environmental variability and geographic distance on variation of bacterial alpha diversity between sites (Euclidean distances). Redundancy analysis (RDA) was used to model the effect of environmental variables on entire microbial communities, with forward selection used to select statistically significant variables. Significance of RDA models and explanatory variables were determined using ANOVAs. Finally, Spearman rank correlations were employed to test associations between environmental parameters on dominant phyla (>1% relative abundance) and ASVs (50 most abundant), with significant correlations visualized using correlation heatmaps.

## Results

After merging, quality filtering, denoising, and removal of chimeric and non-bacterial sequences, a total of 2 435 013 (mean 50 729.44 ± 3303.69) sequences from 48 samples spanning five estuaries were obtained for seawater, 1 712 831 (22 537.25 ± 1004.20) sequences from 75 samples spanning six estuaries were obtained for sediments, and 880 706 (13 761.03 ± 1259.76) sequences from 65 samples spanning six estuaries were obtained for fish hindgut samples.

### Distance-decay patterns

**Figure 2.**
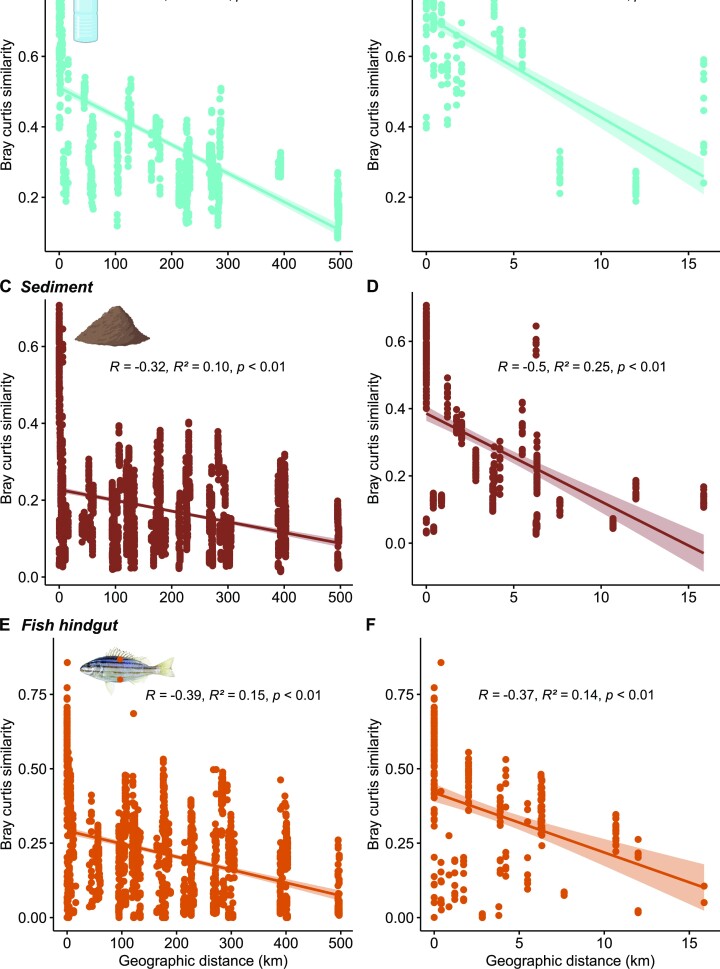
Distance-decay scatterplots showing bacterial similarity (determined by Bray–Curtis similarity) as a function of geographic distance (km) across and within estuaries for seawater (A and B), sediment (C and D), and *Pelates sexlineatus* hindgut (E and F) samples. Created partially with BioRender.com.

Across estuaries, bacterial communities from seawater displayed the strongest distance-decay relationship, with Bray–Curtis similarity decreasing with increasing geographic distance (*R* = −0.69, *P* < 0.01; Fig. 2A). Comparatively weaker relationships were observed for sediment (*R* = −0.32, *P* < 0.01; Fig. 2C) and fish communities (*R* = −0.39, *P* < 0.01; Fig. 2E). Bacterial communities associated with the seawater and sediments exhibited stronger associations between community similarity and geographic distance over smaller spatial scales (within estuaries), and this was strongest for seawater communities (*R* = −0.71, *P* < 0.01; Fig. 2B), followed by sediments (*R* = −0.5, *P* < 0.01; Fig. 2D). Conversely, the distance-decay relationship for fish hindgut samples was slightly weaker within estuaries compared to across estuaries (*R* = −0.37, *P* < 0.01; Fig. 2F). Multiple linear regressions revealed significant associations between seawater community similarity and both geographic distance and environmental variation, as well as a significant interaction between distance and environment across (*P* < 0.01) and within estuaries (*P* < 0.01; Supplementary Table S4). Sediment bacterial communities had significant associations with geographic distance both across and within estuaries; however, environmental variables were only significant at a larger spatial scale (*P* < 0.01; Supplementary Table S4). There was, however, significant interactions between environmental variables and geographic distance at both spatial scales. At both spatial scales, fish hindgut bacterial similarity had a weak association with geographic distance (*P* < 0.01), but not environmental variables (*P* > 0.05); however, there were significant interactions between geographic distance and environmental variables (Supplementary Table S4).

### Bacterial community diversity

**Figure 3.**
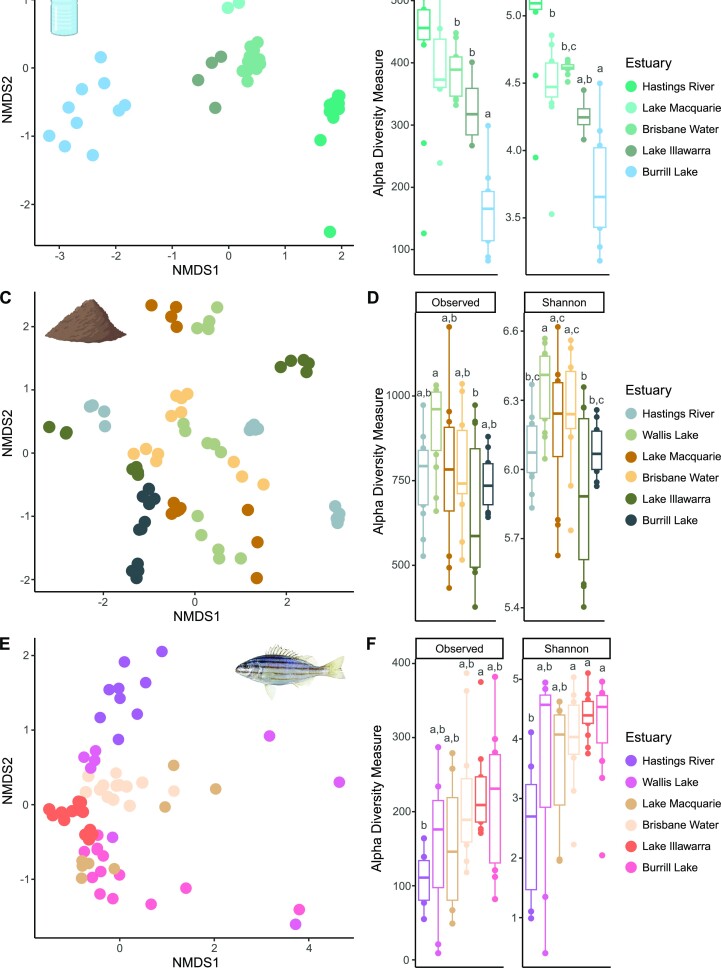
nMDS plots based on Bray–Curtis dissimilarity and alpha diversity metrics (observed richness and Shannon diversity) for seawater (A and B), sediment (C and D), and *Pelates sexlineatus* hindgut (E and F) bacterial communities from each estuary. Created partially with BioRender.com.

Community variance between estuaries was not equal for bacterial communities associated with *P. sexlineatus* hosts (PERMDISP; *F*_5, 57_ = 4.846, *P* = 0.001), seawater (*F*_4, 47_ = 19.844, *P* = 0.001), or sediments (*F*_5, 75_ = 5.7206, *P* = 0.001). Despite this, the results from the PERMANOVA tests and the nMDS plots indicate that for all sample types, bacterial community composition demonstrated significant separation by estuary (*P* < 0.001; Supplementary Table S5), with distinct communities found in each estuary for all sample types (*P* < 0.01; Supplementary Table S6) (Fig. 3A, C, and D). Similarly, alpha diversity (observed richness and Shannon’s diversity) differed significantly between estuaries for each sample type (ANOVA, *P* < 0.01; Supplementary Table S7) (Fig. 3B, D, and F). Seawater communities demonstrated an overall trend of decreasing alpha diversity with decreasing latitude (Figure 3B; Supplementary Table S8). Significant relationships were identified between seawater bacterial alpha diversity and geographic distance (Mantel test, *R* = 0.489, *P* < 0.001), but not environmental factors (*R* = −0.029, *P* = 0.639). Spearman rank correlations were employed to investigate associations between individual environmental variables and seawater bacterial alpha diversity measures, with pH, sediment silt, latitude, average estuary depth, flushing time, catchment area, area of saltmarsh and mangrove in the estuary, and estuary surface area most strongly associated with alpha diversity measures (Supplementary Table S9).

For sediments, observed richness and Shannon diversity were variable across estuaries (Figure 3D; Supplementary Table S8). While significant relationships were not identified between sediment bacterial alpha diversity and geographic distance (*R* = 0.002, *P* = 0.442) or environmental variables (*R* = 0.077, *P* = 0.085), Spearman rank correlations identified significant associations between sediment bacterial alpha diversity measures and particular environmental variables, specifically salinity, pH, sediment organic matter, estuary volume, catchment size, urbanization percentage, and area of seagrass and mangroves (Supplementary Table S9).

For fish hindguts, bacterial alpha diversity generally increased with decreasing latitude (Figure 3F; Supplementary Table S8). Significant relationships were identified between fish hindgut bacterial alpha diversity and geographic distance (*R* = 0.103, *P* = 0.01), but not environmental variables (*R* = 0.038, *P* = 0.280). Significant associations between individual environmental variables and fish hindgut bacterial alpha diversity were identified, particularly sediment silt percentage, latitude, estuary flushing time, catchment area, urbanization percentage, and area of mangroves (Supplementary Table S9).

### Relationships with environmental variables

**Figure 4.**
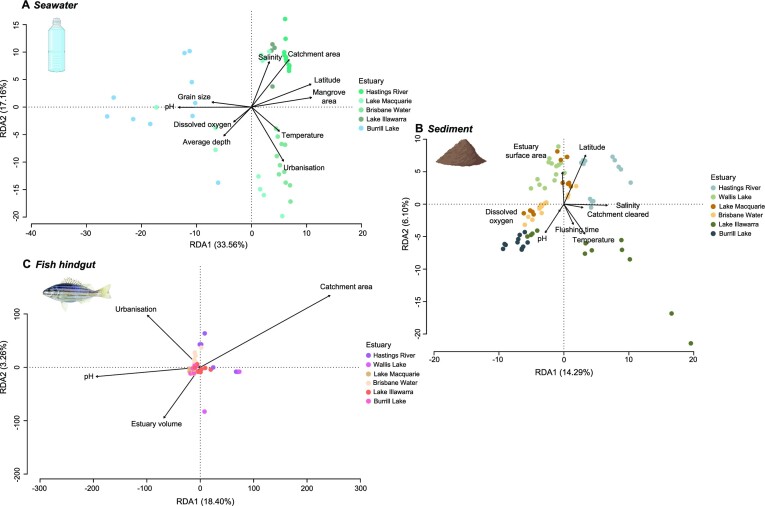
Redundancy analysis (RDA) of environmental variables identified as significant drivers for entire microbial communities associated with (A) seawater, (B) sediment, and (C) *Pelates sexlineatus* hindgut microbiome. Created partially with BioRender.com.

Several environmental variables were identified as significant drivers of variation in seawater and sediment microbial community composition (Figure 4; Supplementary Tables S10 and S11).

Both water column and sediment parameters, along with estuary catchment parameters, were important for free-living communities, explaining 50.72% of total seawater community variation (Fig. 4A) and 20.39% of total sediment community variation (Figure 4B; Supplementary Table S11). This included temperature, salinity, pH, silt, estuary depth, flushing time, and percentage of the catchment i.e. cleared and urbanized (Fig. 4B; Supplementary Table S11). In comparison to seawater and sediment communities, fewer environmental variables were identified as significant drivers of variation in *P. sexlineatus* hindgut microbial communities, with water column pH, catchment area, percentage of urbanization in the estuary catchment, and estuary volume identified as driving 21.66% of the community variation (Figure 4C; Supplementary Table S11).

**Figure 5.**
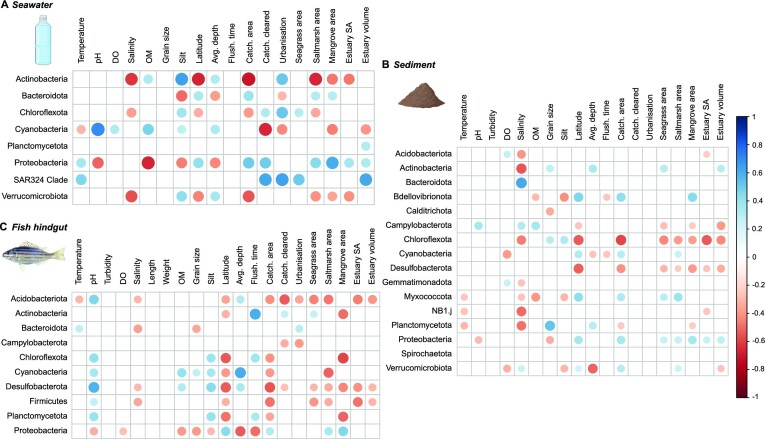
Correlation heatmaps showing Spearman correlations between most abundant phyla (>1% relative abundance in at least one sample) from (A) seawater, (B) sediment, and (C) *Pelates sexlineatus* hindgut samples and environmental variables. Only significant (*P* < 0.05) correlations are shown. Note: DO—dissolved oxygen; OM—sediment organic matter; Temp.—temperature, Flush. time—flushing time, Catch. cleared—percentage of the catchment i.e. cleared, and estuary SA—estuary surface area. Created partially with BioRender.com.

Seawater samples were composed of Proteobacteria (47.33% ± 2.14 relative abundance), Bacteroidota (21.97% ± 1.35), Cyanobacteria (16.76% ± 2.20), Actinobacteria (11.80% ± 0.84), Verrucomicrobiota (0.56% ± 0.08), Planctomycetota (0.37% ± 0.05), Chloroflexota (0.15% ± 0.04), and the SAR324 clade (0.15% ± 0.04) (Fig. 5A). Dominant seawater bacterial phyla showed the strongest correlations with environmental variables in comparison to those from sediment and fish hindgut communities (Fig. 5). Actinobacteria was negatively correlated with salinity, latitude, and catchment area, but positively correlated with sediment silt percentage (Fig. 5A). Proteobacteria was negatively correlated with sediment organic matter, cyanobacteria was positively correlated with pH, and SAR324 was positively correlated with temperature, urbanization (%), estuary volume (ML), and seagrass area (km^2^) (Fig. 5A). Among the most abundant ASVs within seawater samples, members of the Cyanobacteria and Bacteroidota phylum had strong correlations with a number of environmental variables (Supplementary Figure S2). Specifically, within the Cyanobacteria phyla, ASVs identified in the genera *Cyanobium PCC-6307* and *Synechococcus CC902* typically had significant positive correlations with pH, sediment organic matter and silt content, average estuary depth, and flushing time, and negative correlations with latitude (Supplementary Figure S2).

Sediment samples were dominated by Proteobacteria (43.95% ± 0.58), Actinobacteria (18.70% ± 0.87), Bacteroidota (11.36% ± 0.87), Desulfobacterota (6.53% ± 0.32), Chloroflexota (4.80% ± 0.27), Planctomycetota (3.66% ± 0.24), Cyanobacteria (2.76% ± 0.48), Acidobacteriota (2.48% ± 0.15), and Myxococcota (2.49% ± 0.14) (Fig. 5B). Some of the dominant phyla from sediment samples showed strong negative correlations with salinity (Acidobacteriota, Actinobacteria, Chloroflexota, NB1.j, and Planctomycetota) and latitude (Chloroflexota and Desulfobacterota) (Fig. 5B). Bacteroidota was significantly positively correlated with salinity, and some phyla had significant correlations with sediment organic matter, sediment silt (%), and latitude (Fig. 5B). Of the most abundant ASVs recorded in sediment samples, those belonging to the Proteobacteria (genera *Woeseia* and *Methyloceanibacter*) and Actinobacteria phyla (family Propionibacteriaceae and order Actinomarinales) had the strongest associations with environmental variables, particularly negative relationships with water column salinity, latitude, and estuary catchment area (Supplementary Figure S2).

Fish hindgut samples were dominated by Proteobacteria (59.28% ± 2.74), Actinobacteria (20.12% ± 1.82), Cyanobacteria (9.24% ± 1.72), Chloroflexota (5.95% ± 0.52), Firmicutes (2.37% ± 0.57), Planctomycetota (1.43% ± 0.15), Desulfobacterota (0.97% ± 0.25), Campylobacterota (0.27% ± 0.19), Bacteroidota (0.14% ± 0.06), and Acidobacteriota (0.12% ± 0.03) (Fig. 5C). Dominant phyla from fish hindgut samples showed strong negative associations with latitude and estuary catchment area as well as positive correlations with water column pH, sediment silt, and average estuary depth (Fig. 5C). Dominant ASVs associated with the fish gut microbiome had weaker associations with environmental variables than seawater or sediment samples (Supplementary Figure S2). Within the Proteobacteria phylum, ASVs identified as *Methyloceanibacter, Rhodobaculum*, and Rickettsiales had significant associations with pH and salinity, while some ASVs identified as *Synechococcus CC902* and *Cyanobium PCC-6307* (within the Cyanobacteria phylum) had negative associations with latitude (Supplementary Figure S2).

## Discussion

Unveiling the relative importance of spatial and environmental factors in the biogeographic distribution and assembly of microbial communities is a central issue in microbial ecology (Chen et al. 2017, Mo et al. 2018). Studies have focused primarily on free-living communities, but the importance of biogeographic theories for key estuarine host-associated communities are less clear. Here, we demonstrate that while estuarine bacterial communities associated with seawater, sediments, and the hindgut microbiome of a common estuarine fish are shaped across geographic distance, their distributions are shaped by different factors. At the intermediate scale studied here (∼500 km), estuary seawater communities are strongly shaped by both geographic distance and environmental and catchment variables. Despite similarly weak distance-decay relationships for communities associated with the sediments and fish hindguts, sediment communities were influenced by environmental parameters and characterized by greater species richness, potentially reflecting adaptation to steep biogeochemical, and environmental gradients within estuary sediments. On the other hand, the measured environmental variables had minimal influence on overall composition of the fish hindgut microbiome, indicating the importance of host-related factors in maintaining structure of the gut microbiome.

### Geographical distance influences free-living community dissimilarity

Distance-decay relationships for free-living communities are expected to be weak in open ocean systems due to physical mixing resulting in higher dispersal potential and habitat homogeneity (Zinger et al. 2014, Wang et al. 2020). However, coastal environments experience much larger changes in environmental variation than open ocean environments (Zinger et al. 2014), and estuaries act as habitat islands with limited mixing between systems and strong dispersal limitation for bacterial communities across multiple systems (Clark et al. 2021). Here, we demonstrate strong distance-decay relationships for seawater bacterial communities at both intermediate and small spatial scales (i.e. across and within estuaries). At small spatial scales, these patterns may reflect the influence of the increased environmental heterogeneity of coastal environments on bacterial community variation.

The environmental heterogeneity within estuaries is potentially driven by tidal and freshwater exchanges, and therefore water quality variables may be less variable for sediments than seawater. Nevertheless, sediment bacterial communities are likely to be influenced by limited dispersal potential (sessile lifestyles resulting in spatial isolation) and steep biogeochemical gradients within coastal sediments, potentially resulting in strong habitat heterogeneity (Zinger et al. 2014). The sediment communities sampled here displayed weak distance-decay relationships across estuaries, although this relationship became stronger over smaller spatial scales (within estuaries). There are three main processes that could influence these patterns: First, some microorganisms may be dispersal-limited within sediments, which can lead to a decrease in community similarity, as confirmed by a weak but significant decline trend of similarity between sites with increasing geographic distance at both spatial scales (Martiny et al. 2011, Albright and Martiny 2018). Dispersal limitation may also allow for ecological drift of bacterial community composition through stochastic births and deaths and limited dispersal potential, increasing patchiness and partially explaining the stronger distance-decay relationship at a smaller spatial scale (Tuomisto et al. 2003, Martiny et al. 2011). Relic DNA (extracellular DNA from dead microorganisms that may persist in sediments) can also affect community composition and richness, and given that relic DNA is not subject to environmental selection, may reduce distance-decay relationships (Lennon et al. 2018, Clark et al. 2021). Finally, species sorting (adaptation to local environments) may also lead to a decrease in community similarity, as evidenced by significant associations with environmental variables at larger spatial scales (i.e. across estuaries) (Hanson et al. 2012). Environmental selection has been identified as a major process leading to distance-decay relationships and may explain some of the patterns observed here (Hanson et al. 2012).

Another pattern i.e. commonly observed in macroorganisms but still debated in microorganisms is the decline in species richness with increasing latitude (Hillebrand et al. 2004). This trend is stronger across regional scales in comparison to local scales, and increases with organism size and trophic level (Hillebrand et al. 2004). While seawater bacterial communities showed this predicted pattern of higher diversity at lower latitudes, fish hindgut communities demonstrated a weak trend in the opposite direction, and no patterns were detected in sediment communities. The lack of consistent relationships between species richness and latitude here may be a reflection of the relatively small spatial area studied in comparison to previous work that considers much larger latitudinal and environmental gradients (i.e. capturing both temperate and tropical environments) (Hillebrand et al. 2004, Fuhrman et al. 2008). We also note that species richness differed between sample types, lowest in fish hindgut microbiome samples, and greatest in sediments. This may be a reflection of the specialized role of the gut microbiota (Roeselers et al. 2011) and the vast biogeochemical cycles occurring in sediments (Hicks et al. 2018). Higher species richness in sediments may be attributed to steep biogeochemical gradients, higher nutrient concentrations, and higher habitat heterogeneity (Zhao et al. 2020). This increased species richness may also reflect the ability of sediment bacteria to respond to changes in environmental conditions, making this environment more resistant to environmental change (Kerfahi et al. 2014), and supporting the idea that sediment communities studied here are more strongly driven by environmental selection than dispersal limitation. It is important to note that while the Shannon diversity index provides more inferences about community composition and is more robust than simple species richness or evenness by also taking relative abundances of different species into consideration (Kim et al. 2017, Roswell et al. 2021), total microbial diversity was not circumscribed, and thus diversity estimates may not capture all members of the community.

### Environmental factors drive variation in free-living communities

Temperature, salinity, and nutrients have been identified as significant drivers of variation in free-living aquatic microbial communities (Fuhrman et al. 2008, Wietz et al. 2010, Liu et al. 2018, Zhang et al. 2022). At the intermediate scale studied here (∼500 km), we provide evidence that water quality and estuary catchment parameters are important drivers for estuary seawater bacterial communities. Specifically, water column pH and salinity, as well as average depth, flushing time, and percentage of the catchment i.e. cleared, were significant drivers for seawater bacterial community composition and diversity. Genera within the Cyanobacteria, in particular ASVs identified as *Synechococcus CC9902* and *Cyanobium PCC-6307*, were associated with increased pH, decreased salinity, and smaller catchment areas with decreased saltmarsh and mangrove areas. These genera are important primary producers in open ocean environments and have been frequently associated with blooms in coastal areas, particularly after storms or large freshwater inputs (Xia et al. 2015, Li et al. 2019). They have also been associated with other abundant taxa reported here, including *Candidatus Actinomarina*, HIMB11*, Winogradskyella*, and NS5 marine group, which were also found associated with similar environmental variables (Li et al. 2019, Fortin et al. 2022).

For sediment communities, significant associations between community similarity and environmental variables were only documented at larger spatial scales, indicating that these communities may be less strongly influenced by dispersal than environmental filtering across larger spatial scales. Here, salinity was most frequently associated with sediment microbial community diversity and composition, consistent with findings from other systems (Webster et al. 2015, Vekeman et al. 2016, Huang et al. 2019, Yue et al. 2022). The most abundant ASVs belonging to taxa within the Proteobacteria (*Methyloceanibacter* and *Woeseia*) and Actinobacteria (Propionibacteriaceae and Actinomarinales) were significantly correlated with salinity and are abundant in estuarine sediments, likely carrying out diverse ecological functions including denitrification, sulfur oxidation, and aerobic ammonium oxidation (Vekeman et al. 2016, Mußmann et al. 2017, Rios-Del Toro et al. 2018, Zhang et al. 2020). Previous findings also indicate, however, that there is a large amount of unexplained variation in these communities, consistent with findings here. These studies suggest that this is a result of either unmeasured environmental variability or stochastic processes such as ecological drift or random speciation and extinction (Martiny et al. 2011, Xiong et al. 2014, Yue et al. 2022, Zhang et al. 2022).

### Pelates sexlineatus hindgut microbiome is governed by host control

Previous work on fish has revealed that both environmental and host-related factors are important in shaping the hindgut microbiome (Sullam et al. 2012, Stephens et al. 2016, Tarnecki et al. 2017). Studies investigating the influence of spatial factors on the fish gut microbiome are limited; however, one study found that community composition was not significantly impacted by geography and instead host-related factors, particularly life stage, strongly defined community composition, and diversity (Llewellyn et al. 2016). While alpha diversity generally increased with decreasing latitude, distance-decay relationships were weak across both spatial scales examined here, and associations with environmental variables were generally limited and weak, suggesting that host-related factors, rather than spatial or environmental factors, play a primary role in structuring and maintaining *P. sexlineatus* hindgut bacterial communities. Previous work has also highlighted the importance of host-related factors rather than the external environment in driving shifts in the gut microbiome of fish, e.g. host phylogeny (Sullam et al. 2012) and gut physiology (Stephens et al. 2016). Host genetics may also influence the gut microbiome (Kokou et al. 2018), and genetic structure of dispersal-limited organisms has been shown to correlate with geography (e.g. algae) (Wood et al. 2022). *Pelates sexlineatus* is thought to spawn near the mouth of estuaries, with juveniles moving into and remaining in estuaries for at least one year after settlement; therefore, dispersal may be limited to an individual estuary (Smith and Suthers 2000), and may explain the weaker distance-decay relationships on a smaller spatial scale (within estuaries). It is important to note, however, that we did not measure the age of individuals nor the internal environmental conditions of the fish hindgut, and these may have important driving forces on these communities (Li et al. 2020), limiting our ability to draw conclusions on the buffering effect of the host. Future work should consider the effect of both the external and internal environment on the fish gut microbiome in order to further disentangle the responses of these communities to spatial and environmental influences.

While many of the dominant ASVs reported here are common marine and estuarine environmental taxa (e.g. *Methyloceaniabcter, Cyanobium PCC-6307*, and *Synechococcus CC9902*), ASVs belonging to the bacterial families Propionibacteriaceae, Rhodobacteraceae, and Vibrionaceae were also abundant, consistent with previous findings (Larios‐Soriano et al. 2021, Suzzi et al. 2022). The Propionibacteriaceae produce microbial metabolites during glucose fermentation and enzymes for fatty acid degradation (Neis et al. 2015, Chapagain et al. 2019) that may help in the breakdown of food, produce valuable nutrients, and energy. Relationships between abundance of the Propionibacteriaceae and host diet have been established (Larios‐Soriano et al. 2021), and given that *P. sexlineatus* is an opportunistic carnivore that feeds on dominant food sources within seagrass meadows, environmental filtering may play an important role in structuring and maintaining the gut microbiome across estuaries through diet; however, this was not measured here (Sanchez‐Jerez et al. 2002, Loo et al. 2019).

## Conclusion

A central goal in microbial ecology is understanding the contribution of spatial and environmental factors in driving patterns of microbial composition and diversity. Biogeography has been shown to apply to free-living microbial communities across interconnected aquatic environments, with the relative importance of specific environmental variables identified at a range of spatial scales. Patterns in free-living bacterial communities are likely to differ from those in host-associated communities, which may be buffered from environmental variation and under stronger selective pressure from the niche habitat provided by the host and are comparatively less well understood. We found that spatial and environmental factors have different influences on the bacterial communities associated with estuary seawater, sediments, and a common estuarine fish, *P. sexlineatus*, across six eastern Australian estuaries spanning ∼500 km. Seawater communities exhibited strong distance-decay relationships as well as significant associations with a range of environmental variables. Conversely, sediment and *P. sexlineatus* hindgut bacterial communities displayed weak distance-decay relationships and limited variation explained by measured environmental variables, potentially reflecting environmental filtering across biogeochemical gradients or stochastic processes within estuary sediments and that host-associated communities are governed most strongly by host-related factors. These results provide important ecological insights into the spatial distribution and some of the driving factors of bacterial community composition across temperate estuarine systems for multiple sample types.

## Supplementary Material

fiad061_Supplemental_Files

## Data Availability

The sequence data from seawater and sediment samples are freely available at the BioPlatforms Australia data portal under the Australian Microbiome project (DOI: 102.100/401 931) ([dataset]* 2022). Sequence data from fish hindgut samples have been submitted to the NCBI Sequence Read Archive (SRA) under Bioproject number PRJNA912232 ([dataset]* Suzzi et al. 2022).
