# Supplementary material for: Spatial patterns in host-associated and free-living bacterial communities across six temperate estuaries
Source: FEMS Microbiol Ecol. 2023 Jun 2;99(7):fiad061. doi: 10.1093/femsec/fiad061 (PMC10284270; doi:10.1093/femsec/fiad061)
Supplement: fiad061_Supplemental_Files [file fiad061_supplemental_files.zip › Suzzi_FEMS_Supp_data Tables.docx]

Table S1. Summary of samples successfully sequenced across estuary, sample site and sample type, before and after rarefaction to 4,276 reads.

| **Estuary** | **Site** | **Fish samples before rarefaction** | **Fish samples after rarefaction** | **Sediment samples before rarefaction** | **Sediment samples after rarefaction** | **Seawater samples before rarefaction** | **Seawater samples after rarefaction** |
| --- | --- | --- | --- | --- | --- | --- | --- |
| Burrill Lake | Site 1 | 5 | 5 | 4 | 4 | 4 | 4 |
|  | Site 2 | 3 | 2 | 3 | 3 | 3 | 3 |
|  | Site 3 | 5 | 5 | 4 | 4 | 3 | 3 |
| Brisbane Water | Saratoga | 5 | 5 | 3 | 3 | 4 | 4 |
|  | Green Point | 3 | 2 | 4 | 4 | 4 | 4 |
|  | Woy Woy | 5 | 4 | 5 | 5 | 3 | 3 |
| Hastings River | Site 1 | 4 | 3 | 5 | 5 | 4 | 4 |
|  | Site 2 | 3 | 3 | 4 | 4 | 5 | 5 |
|  | Site 3 | 2 | 1 | 5 | 5 | 3 | 3 |
| Lake Illawarra | Site 1 | 4 | 4 | 4 | 4 | 0 | 0 |
|  | Site 2 | 5 | 5 | 5 | 5 | 4 | 4 |
|  | Site 3 | 4 | 4 | 3 | 3 | 0 | 0 |
| Lake Macquarie | Belmont | 5 | 5 | 5 | 5 | 4 | 4 |
|  | Fennell Bay | 1 | 1 | 4 | 4 | 3 | 3 |
|  | Gwandalan | 2 | 2 | 3 | 3 | 4 | 4 |
| Wallis Lake | Green Point | 3 | 1 | 5 | 5 | 2 | 0 |
|  | Mosquito Island | 4 | 4 | 5 | 5 | 0 | 0 |
|  | Tonys Point | 2 | 2 | 5 | 5 | 0 | 0 |
| **TOTALS** |  | **65** | **58** | **75** | **75** | **50** | **48** |

Table S2. Environmental variables used in this study. Temperature (Temp.), pH, turbidity and dissolved oxygen (DO) were recorded in the field using a Horiba U-50 water quality meter, and sediment organic matter (OM), grain size and silt (%) (mean ± SE, n = 3) were determined following sample collection. Average depth, flushing time, catchment area, catchment cleared, urbanisation, estuary surface area (SA) and estuary volume were extracted from the NSW Estuary Health Monitoring, Evaluating and Reporting (MER) program on the SEED NSW database (Department of Planning and Environment, 2020). Seagrass, mangrove and saltmarsh area was extracted from NSW estuarine macrophyte data, obtained from the Fisheries NSW Spatial Data Portal (NSW Department of Primary Industries).

| **Estuary** | **Site** | **Temp.** | **pH** | **Turbidity** | **DO** | **Salinity** | **OM** | **Grain size** | **Silt** | **Latitude** | **Average depth (m)** | **Flushing time (days)** | **Catchment area (km^2^)** | **Catchment cleared (%)** | **Urbanisation (%)** | **Estuary SA (km^2^)** | **Estuary volume (ML)** | **Seagrass area (km^2^)** | **Saltmarsh area (km^2^)** | **Mangrove area (km^2^)** |
| --- | --- | --- | --- | --- | --- | --- | --- | --- | --- | --- | --- | --- | --- | --- | --- | --- | --- | --- | --- | --- |
| **Burrill Lake** | Site 1 | 20.60 | 8.25 | - | 8.69 | 26.13 | 1.77 ± 0.41 | 763.9 ± 255.4 | 6.94 ± 0.88 | -35.3697 | 4.3 | 44.46 | 78.0 | 12.52 | 3.00 | 4.4 | 17652.70 | 0 | 0.11 | 0 |
|  | Site 2 | 21.50 | 8.40 | - | 11.39 | 26.95 | 2.13 ± 0.50 | 580.6 ± 24.0 | 9.43 ± 0.48 | -35.38045 | 4.3 | 44.46 | 78.0 | 12.52 | 3.00 | 4.4 | 17652.70 | 0 | 0.11 | 0 |
|  | Site 3 | 22.78 | 8.27 | - | 7.95 | 26.29 | 2.91 ± 1.05 | 611.9 ± 241.7 | 7.98 ± 0.77 | -35.3863 | 4.3 | 44.46 | 78.0 | 12.52 | 3.00 | 4.4 | 17652.70 | 0 | 0.11 | 0 |
| **Brisbane Water** | Green Point | 22.67 | 6.90 | - | 5.09 | 24.9 | 1.73 ± 0.83 | 352.7 ± 32.6 | 7.56 ± 1.22 | -33.4511 | 3.1 | 24.56 | 152.55 | 52.13 | 46.35 | 28.3 | 84198.69 | 5.58 | 27.22 | 2.08 |
|  | Saratoga | 23.48 | 7.06 | 1.8 | 6.56 | 25.6 | 0.17 ± 0.01 | 273.1 ± 9.48 | 7.01 ± 1.66 | -33.4881 | 3.1 | 24.56 | 152.55 | 52.13 | 46.35 | 28.3 | 84198.69 | 5.58 | 27.22 | 2.08 |
|  | Woy Woy | 26.87 | 7.03 | 22.2 | 9.49 | 24.0 | 0.16 ± 0.01 | 280.6 ± 6.23 | 4.72 ± 0.33 | -33.4838 | 3.1 | 24.56 | 152.55 | 52.13 | 46.35 | 28.3 | 84198.69 | 5.58 | 27.22 | 2.08 |
| **Hastings River** | Site 1 | 21.53 | 6.79 | 3.0 | 9.73 | 29.0 | 0.26 ± 0.09 | 325.8 ± 3.55 | 4.14 ± 0.13 | -31.43 | 1.9 | 12.73 | 3658.57 | 30.25 | 2.45 | 30.0 | 52685.86 | 1.46 | 28.09 | 3.44 |
|  | Site 2 | 22.44 | 7.08 | 1.9 | 6.85 | 29.7 | 0.43 ± 0.00 | 314.9 ± 20.9 | 3.45 ± 0.00 | -31.4286 | 1.9 | 12.73 | 3658.57 | 30.25 | 2.45 | 30.0 | 52685.86 | 1.46 | 28.09 | 3.44 |
|  | Site 3 | 22.81 | 7.14 | 0.6 | 7.77 | 30.3 | 0.29 ± 0.05 | 281.0 ± 18.4 | 3.43 ± 0.13 | -31.4254 | 1.9 | 12.73 | 3658.57 | 30.25 | 2.45 | 30.0 | 52685.86 | 1.46 | 28.09 | 3.44 |
| **Lake Illawarra** | Site 1 | 25.23 | 7.03 | 2.3 | 8.81 | 27.0 | 0.42 ± 0.16 | 377.5 ± 31.9 | 5.83 ± 0.38 | -34.4925 | 2.1 | 260.69 | 238.43 | 59.34 | 21.81 | 35.8 | 74275.06 | 7.97 | 35.53 | 0 |
|  | Site 2 | 25.39 | 7.35 | 6.9 | 7.68 | 30.6 | 0.55 ± 0.05 | 331.4 ± 111.7 | 6.46 ± 0.45 | -34.5011 | 2.1 | 260.69 | 238.43 | 59.34 | 21.81 | 35.8 | 74275.06 | 7.97 | 35.53 | 0 |
|  | Site 3 | 26.73 | 7.42 | 42.2 | 8.50 | 31.0 | 2.78 ± 0.28 | 194.0 ± 7.24 | 11.92 ± 0.10 | -34.5602 | 2.1 | 260.69 | 238.43 | 59.34 | 21.81 | 35.8 | 74275.06 | 7.97 | 35.53 | 0 |
| **Lake Macquarie** | Belmont | 21.94 | 7.06 | 0 | 10.44 | 28.3 | 0.67 ± 0.11 | 435.3 ± 38.0 | 4.50 ± 0.41 | -33.0462 | 8.0 | 249.86 | 604.39 | 40.08 | 19.7 | 110.0 | 669190.0 | 12.79 | 0.78 | 1.59 |
|  | Fennell Bay | 23.02 | 7.33 | 2.6 | 10.0 | 29.6 | 1.24 ± 0.00 | 238.3 ± 1.27 | 6.62 ± 0.00 | -32.9947 | 8.0 | 249.86 | 604.39 | 40.08 | 19.7 | 110.0 | 669190.0 | 12.79 | 0.78 | 1.59 |
|  | Gwandalan | 22.31 | 7.25 | 3.9 | 11.82 | 29.6 | 1.00 ± 0.03 | 597.5 ± 125.3 | 5.02 ± 0.17 | -33.1419 | 8.0 | 249.86 | 604.39 | 40.08 | 19.7 | 110.0 | 669190.0 | 12.79 | 0.78 | 1.59 |
| **Wallis Lake** | Green Point | 20.13 | 7.36 | 3.7 | 4.07 | 26.1 | 0.49 ± 0.00 | 348.2 ± 6.75 | 3.22 ± 0.00 | -32.2408 | 2.35 | 76.03 | 1196.90 | 45.07 | 6.06 | 98.7 | 217951.5 | 31.9 | 92.8 | 1.47 |
|  | Mosquito Island | 22.45 | 7.81 | 3.3 | 7.42 | 27.2 | 0.31 ± 0.07 | 295.2 ± 21.7 | 5.11 ± 0.36 | -32.196 | 2.35 | 76.03 | 1196.9 | 45.07 | 6.06 | 98.7 | 217951.5 | 31.9 | 92.8 | 1.47 |
|  | Tonys Point | 21.26 | 6.19 | 2.1 | 6.56 | 27.5 | 0.06 ± 0.02 | 366.2 ± 23.6 | 4.53 ± 0.09 | -32.2101 | 2.35 | 76.03 | 1196.9 | 45.07 | 6.06 | 98.7 | 217951.5 | 31.9 | 92.8 | 1.47 |

Table S3. Output from linear models assessing environmental variables (based on Euclidean distances) against geographic distance.

|  | **Estimate** | **Std Error** | **T** | **P** |
| --- | --- | --- | --- | --- |
| Intercept | 171.97861 | 6.18791 | 27.79 | ***<0.001*** |
| Geographic distance | 0.42109 | 0.02623 | 16.69 | ***<0.001*** |

Table S4. Output from linear models comparing seawater, sediment and fish hindgut bacterial similarity with geographic distance and all environmental parameters (temperature, salinity, pH, turbidity, dissolved oxygen, sediment organic matter, sediment grain size, average depth, flushing time, catchment area, catchment cleared, urbanisation, estuary surface area, estuary volume and seagrass, mangrove and saltmarsh area) both across and within estuaries.

|  | **Estimate** | **Std Error** | **T** | **P** |
| --- | --- | --- | --- | --- |
| **Across Estuaries** |  |  |  |  |
| **Seawater (R^2^ = 0.56)** |  |  |  |  |
| Intercept | 6.034e-01 | 8.622e-03 | 69.99 | ***<0.001*** |
| Distance | -1.122e-03 | 3.527e-05 | -31.81 | ***<0.001*** |
| Environment | -3.707e-04 | 2.587e-05 | -14.33 | ***<0.001*** |
| Distance*Environment | 1.185e-06 | 8.902e-08 | 13.31 | ***<0.001*** |
| **Sediment (R^2^ = 0.12)** |  |  |  |  |
| Intercept | 2.501e-01 | 5.691e-03 | 43.949 | ***<0.001*** |
| Distance | -4.047e-04 | 2.531e-05 | -15.989 | ***<0.001*** |
| Environment | -1.239e-04 | 2.041e-05 | -6.073 | ***<0.001*** |
| Distance*Environment | 5.278e-07 | 7.314e-08 | 7.216 | ***<0.001*** |
| **Fish hindgut (R^2^ = 0.17)** | | | | |
| Intercept | 3.094e-01 | 1.003e-02 | 30.867 | ***<0.001*** |
| Distance | -6.172e-04 | 4.640e-05 | -13.302 | ***<0.001*** |
| Environment | -3.483e-05 | 3.477e-05 | -1.002 | 0.316 |
| Distance*Environment | 5.176e-07 | 1.420e-07 | 3.646 | ***<0.001*** |
| **Within Estuary** |  |  |  |  |
| **Seawater (R^2^ = 0.62)** |  |  |  |  |
| Intercept | 7.681e-01 | 1.135e-02 | 67.673 | ***<0.001*** |
| Distance | -4.042e-02 | 2.769e-03 | -14.600 | ***<0.001*** |
| Environment | -3.414e-04 | 4.326e-05 | -7.892 | ***<0.001*** |
| Distance*Environment | 5.412e-05 | 8.692e-06 | 6.226 | ***<0.001*** |
| **Sediment (R^2^ = 0.28)** |  |  |  |  |
| Intercept | 3.809e-01 | 1.386e-02 | 27.473 | ***<0.001*** |
| Distance | -3.614e-02 | 3.587e-03 | -10.075 | ***<0.001*** |
| Environment | 1.040e-04 | 6.814e-05 | 1.527 | 0.127 |
| Distance*Environment | 3.609e-05 | 1.309e-05 | 2.758 | ***0.006*** |
| **Fish hindgut (R^2^ = 0.11)** | |  |  |  |
| Intercept | 4.152e01 | 2.157e-02 | 19.250 | ***<0.001*** |
| Distance | -2.753e-02 | 5.881e-03 | -4.681 | ***<0.001*** |
| Environment | -1.990e-05 | 8.484e-05 | -0.235 | 0.815 |
| Distance*Environment | 8.636e-05 | 1.979e-05 | 4.363 | ***<0.001*** |

Table S5. Summary of PERMANOVA output (based on Bray-Curtis dissimilarities) comparing bacterial communities associated with each sample type across estuaries.

|  | **Df** | **Sum Sq** | **Mean Sq** | **F** | **R^2^** | **P** |
| --- | --- | --- | --- | --- | --- | --- |
| **Seawater** |  |  |  |  |  |  |
| Estuary | 4 | 6.9236 | 1.73091 | 19.844 | 0.64863 | ***0.001*** |
| Residuals | 43 | 3.7506 | 0.08722 |  | 0.35137 |  |
| Total | 47 | 10.6743 |  |  | 1.0000 |  |
| **Sediment** |  |  |  |  |  |  |
| Estuary | 5 | 7.595 | 1.51901 | 5.7206 | 0.29008 | ***0.001*** |
| Residuals | 70 | 18.587 | 0.26554 |  | 0.70992 |  |
| Total | 75 | 26.183 |  |  | 1.00000 |  |
| **Fish hindgut** |  |  |  |  |  |  |
| Estuary | 5 | 5.8858 | 1.17716 | 4.8466 | 0.31788 | ***0.001*** |
| Residuals | 52 | 12.6301 | 0.24289 |  | 0.68212 |  |
| Total | 57 | 18.5159 |  |  | 1.00000 |  |

Table S6. Summary of PERMANOVA pairwise comparisons (based on Bray-Curtis dissimilarities) comparing bacterial communities associated with each sample type across estuaries.

|  | **Brisbane Water** | **Burrill**  **Lake** | **Hastings River** | **Lake Illawarra** | **Lake Macquarie** |
| --- | --- | --- | --- | --- | --- |
| **Seawater** |  |  |  |  |  |
| Burrill Lake | ***0.001*** |  |  |  |  |
| Hastings River | ***0.001*** | ***0.001*** |  |  |  |
| Lake Illawarra | ***0.001*** | ***0.002*** | ***0.001*** |  |  |
| Lake Macquarie | ***0.001*** | ***0.001*** | ***0.001*** | ***0.006*** |  |
| **Sediment** |  |  |  |  |  |
| Burrill Lake | ***0.001*** |  |  |  |  |
| Hastings River | ***0.001*** | ***0.001*** |  |  |  |
| Lake Illawarra | ***0.001*** | ***0.001*** | ***0.004*** |  |  |
| Lake Macquarie | ***0.001*** | ***0.001*** | ***0.001*** | ***0.001*** |  |
| Wallis Lake | ***0.001*** | ***0.001*** | ***0.001*** | ***0.001*** | ***0.001*** |
| **Fish hindgut** |  |  |  |  |  |
| Burrill Lake | ***0.022*** |  |  |  |  |
| Hastings River | ***0.002*** | ***0.018*** |  |  |  |
| Lake Illawarra | ***0.001*** | ***0.001*** | ***0.001*** |  |  |
| Lake Macquarie | ***0.001*** | ***0.001*** | ***0.002*** | ***0.001*** |  |
| Wallis Lake | ***0.001*** | ***0.001*** | ***0.009*** | ***0.001*** | ***0.001*** |

Table S7. Summary of ANOVA output comparing alpha diversity (observed richness and Shannon’s diversity) across estuary for each sample type.

|  | Df | Sum sq | Mean sq | F | P |
| --- | --- | --- | --- | --- | --- |
| **Seawater** |  |  |  |  |  |
| **Observed** |  |  |  |  |  |
| Estuary | 4 | 459101 | 114775 | 17.81 | ***<0.001*** |
| Residuals | 43 | 277108 | 6444 |  |  |
| **Shannon** |  |  |  |  |  |
| Estuary | 4 | 8.938 | 2.235 | 21.7 | ***<0.001*** |
| Residuals | 43 | 4.428 | 0.103 |  |  |
| **Sediment** |  |  |  |  |  |
| **Observed** |  |  |  |  |  |
| Estuary | 5 | 492567 | 98513 | 3.929 | ***0.003*** |
| Residuals | 70 | 1755343 | 25076 |  |  |
| **Shannon** |  |  |  |  |  |
| Estuary | 5 | 1.764 | 0.3529 | 6.833 | ***<0.001*** |
| Residuals | 70 | 3.615 | 0.0516 |  |  |
| **Fish hindgut** |  |  |  |  |  |
| **Observed** |  |  |  |  |  |
| Estuary | 5 | 94390 | 18878 | 2.897 | ***0.022*** |
| Residuals | 52 | 338888 | 6517 |  |  |
| **Shannon** |  |  |  |  |  |
| Estuary | 5 | 20.17 | 4.034 | 3.894 | ***0.004*** |
| Residuals | 52 | 53.87 | 1.036 |  |  |

Table S8. Summary of Tukey’s HSD output comparing alpha diversity (observed richness and Shannon’s diversity) across estuary for each sample type.

|  | **Brisbane Water** | **Burrill**  **Lake** | **Hastings River** | **Lake Illawarra** | **Lake Macquarie** |
| --- | --- | --- | --- | --- | --- |
| **Seawater** |  |  |  |  |  |
| **Observed** |  |  |  |  |  |
| Burrill Lake | ***<0.001*** |  |  |  |  |
| Hastings River | 0.717 | ***<0.001*** |  |  |  |
| Lake Illawarra | 0.726 | ***0.011*** | 0.211 |  |  |
| Lake Macquarie | 0.994 | ***<0.001*** | 0.911 | 0.548 |  |
| **Shannon** |  |  |  |  |  |
| Burrill Lake | ***<0.001*** |  |  |  |  |
| Hastings River | 0.067 | ***<0.001*** |  |  |  |
| Lake Illawarra | 0.331 | 0.060 | ***<0.001*** |  |  |
| Lake Macquarie | 0.784 | ***<0.001*** | ***0.003*** | 0.821 |  |
| **Sediment** |  |  |  |  |  |
| **Observed** |  |  |  |  |  |
| Burrill Lake | 0.989 |  |  |  |  |
| Hastings River | 0.998 | 0.999 |  |  |  |
| Lake Illawarra | 0.320 | 0.730 | 0.512 |  |  |
| Lake Macquarie | 0.999 | 0.997 | 0.999 | 0.430 |  |
| Wallis Lake | 0.285 | 0.082 | 0.100 | ***<0.001*** | 0.196 |
| **Shannon** |  |  |  |  |  |
| Burrill Lake | 0.562 |  |  |  |  |
| Hastings River | 0.457 | 0.999 |  |  |  |
| Lake Illawarra | ***0.003*** | 0.307 | 0.274 |  |  |
| Lake Macquarie | 0.970 | 0.994 | 0.909 | ***0.036*** |  |
| Wallis Lake | 0.710 | ***0.028*** | ***0.013*** | ***<0.001*** | 0.231 |
| **Fish hindgut** |  |  |  |  |  |
| **Observed** |  |  |  |  |  |
| Burrill Lake | 0.999 |  |  |  |  |
| Hastings River | 0.076 | 0.076 |  |  |  |
| Lake Illawarra | 0.999 | 0.999 | ***0.041*** |  |  |
| Lake Macquarie | 0.558 | 0.572 | 0.878 | 0.416 |  |
| Wallis Lake | 0.636 | 0.651 | 0.871 | 0.501 | 0.999 |
| **Shannon** |  |  |  |  |  |
| Burrill Lake | 0.998 |  |  |  |  |
| Hastings River | ***0.032*** | ***0.009*** |  |  |  |
| Lake Illawarra | 0.920 | 0.993 | ***0.001*** |  |  |
| Lake Macquarie | 0.955 | 0.804 | 0.275 | 0.486 |  |
| Wallis Lake | 0.959 | 0.821 | 0.317 | 0.522 | 1.000 |

Table S9. Spearman rank correlations between seawater, sediment and fish hindgut alpha diversity measures and environmental variables.

|  | **Seawater** | | **Sediment** | | **Fish hindgut** | |
| --- | --- | --- | --- | --- | --- | --- |
|  | **Observed** | **Shannon** | **Observed** | **Shannon** | **Observed** | **Shannon** |
| **Temperature** | R = -0.03  p = 0.82 | R = -0.03  p = 0.83 | R = -0.20  p = 0.07 | R = -0.18  p = 0.10 | R = 0.19  p = 0.16 | R = 0.11  p = 0.41 |
| **Salinity** | R = 0.24  p = 0.10 | R = 0.12  p = 0.39 | ***R = -0.32***  ***p < 0.01*** | ***R = -0.42***  ***p < 0.01*** | R = -0.22  p = 0.09 | R = -0.15  p = 0.25 |
| **pH** | ***R = -0.58***  ***p = < 0.01*** | ***R = -0.68***  ***p < 0.01*** | ***R = -0.20***  ***p = 0.08*** | ***R = -0.29***  ***p = 0.01*** | R = 0.22  p = 0.09 | ***R = 0.37***  ***p = <0.01*** |
| **Dissolved oxygen** | R < 0.00  p = 0.99 | R = -0.11  p = 0.43 | R = -0.08  p = 0.44 | R = -0.07  p = 0.53 | R = -0.12  p = 0.34 | R = -0.17  p = 0.18 |
| **Organic matter** | R = -0.23  p = 0.11 | ***R = -0.40***  ***p < 0.01*** | ***R = -0.47***  ***p < 0.01*** | ***R = -0.50***  ***p < 0.01*** | R = -0.02  p = 0.87 | R = 0.03  p = 0.77 |
| **Grain size** | R = -0.17  p = 0.24 | R = -0.17  p = 0.22 | R = 0.04  p = 0.68 | R = 0.03  p = 0.78 | R = -0.07  p = 0.55 | R = -0.04  p = 0.71 |
| **Silt** | ***R = -0.48***  ***p < 0.01*** | ***R = -0.59***  ***p < 0.01*** | ***R = -0.25***  ***p = 0.02*** | R = -0.19  p = 0.08 | ***R = 0.25***  ***p = 0.05*** | ***R = 0.27***  ***p = 0.03*** |
| **Latitude** | ***R = 0.64***  ***p < 0.01*** | ***R = 0.64***  ***p < 0.01*** | ***R = 0.25***  ***p = 0.02*** | R = 0.20  p = 0.07 | ***R = -0.38***  ***p < 0.01*** | ***R = -0.35***  ***p < 0.01*** |
| **Average estuary depth** | ***R = -0.29***  ***p = 0.04*** | ***R = -0.49***  ***p < 0.01*** | R = 0.04  p = 0.72 | R = 0.18  p = 0.10 | R = 0.09  p = 0.48 | R = 0.38  p = 0.11 |
| **Flushing time** | ***R = -0.33***  ***p = 0.02*** | ***R = -0.58***  ***p < 0.01*** | R = -0.06  p = 0.55 | R = -0.02  p = 0.83 | R = 0.23  p = 0.07 | ***R = 0.29***  ***p = 0.02*** |
| **Catchment area** | ***R = 0.66 p < 0.01*** | ***R = 0.64***  ***p < 0.01*** | R = 0.17 p = 0.12 | R = 0.08 p = 0.44 | ***R = -0.36 p < 0.01*** | ***R = -0.31 p = 0.01*** |
| **Catchment cleared** | R = 0.25 p = 0.07 | R = 0.17 p = 0.22 | R = -0.01  P = 0.95 | R = 0.08 p = 0.48 | R = 0.20 p = 0.11 | R = 0.14 p = 0.27 |
| **Urbanisation** | R = -0.09  p = 0.51 | R = -0.16  p = 0.26 | R = -0.05  p = 0.64 | R = 0.10  p = 0.34 | ***R = 0.28***  ***p = 0.03*** | R = 0.17  p = 0.17 |
| **Seagrass area** | R = -0.03 p = 0.79 | ***R = -0.27 p = 0.05*** | ***R = 0.28 p = 0.01*** | ***R = 0.36 p < 0.01*** | R = -0.03 p = 0.78 | R = 0.03  p = 0.76 |
| **Saltmarsh area** | ***R = 0.68 p < 0.01*** | ***R = 0.71 p < 0.01*** | R = 0.21 p = 0.06 | R = 0.16 p = 0.15 | R = -0.03 p = 0.80 | R = 0.01 p = 0.92 |
| **Mangrove area** | ***R = 0.66 p < 0.01*** | ***R = 0.80 p < 0.01*** | R = 0.08 p = 0.45 | R = 0.09 p = 0.39 | ***R = -0.34 p < 0.01*** | ***R = -0.42 p < 0.01*** |
| **Estuary surface area** | ***R = 0.48***  ***p < 0.01*** | ***R = 0.28***  ***p = 0.05*** | R = 0.16  p = 0.16 | R = 0.20  p = 0.07 | R = -0.15  p = 0.25 | R = -0.08  p = 0.53 |
| **Estuary volume** | ***R = 0.38 p < 0.01*** | R = 0.21 p = 0.13 | ***R = 0.24 p = 0.03*** | ***R = 0.38 p < 0.01*** | R = -0.08 p = 0.54 | R = -0.06 p = 0.65 |
| **Fish length** |  |  |  |  | R = -0.05  p = 0.69 | R = 0.02  p = 0.87 |

Table S10. Summary of ANOVA output testing significance of RDA models on bacterial communities associated with seawater, sediment and fish hindguts.

|  | **df** | **Variance** | **F** | **P** |
| --- | --- | --- | --- | --- |
| **Seawater** |  |  |  |  |
| Model | 10 | 246842 | 13.419 | ***0.001*** |
| Residuals | 37 | 68059 |  |  |
| **Sediment** |  |  |  |  |
| Model | 8 | 22238 | 5.6786 | ***0.001*** |
| Residual | 67 | 32798 |  |  |
| **Fish hindgut** |  |  |  |  |
| Model | 4 | 422200 | 4.1071 | ***0.001*** |
| Residuals | 50 | 1284958 |  |  |

Table S11. Summary of ANOVA output testing significance of variables included in RDA models for bacterial seawater, sediment and fish hindgut communities.

|  | **df** | **Variance** | **F** | **P** |
| --- | --- | --- | --- | --- |
| **Seawater** |  |  |  |  |
| Temperature | 1 | 25274 | 13.7401 | ***0.001*** |
| Salinity | 1 | 31022 | 16.8652 | ***0.001*** |
| Latitude | 1 | 62198 | 33.8139 | ***0.001*** |
| pH | 1 | 30073 | 16.3490 | ***0.001*** |
| Dissolved oxygen | 1 | 16222 | 8.8189 | ***0.001*** |
| Sediment grain size | 1 | 9646 | 5.2440 | ***0.001*** |
| Average depth (m) | 1 | 20296 | 11.0340 | ***0.001*** |
| Mangrove area (km^2^) | 1 | 29360 | 15.9614 | ***0.001*** |
| Catchment area (km^2^) | 1 | 9459 | 5.1422 | ***0.001*** |
| Urbanisation (%) | 1 | 13291 | 7.2258 | ***0.001*** |
| Residuals | 37 | 68059 |  |  |
| **Sediment** |  |  |  |  |
| Temperature | 1 | 3106 | 6.3455 | ***0.001*** |
| Salinity | 1 | 5391 | 11.0127 | ***0.001*** |
| Latitude | 1 | 3436 | 7.0184 | ***0.001*** |
| pH | 1 | 1431 | 2.9242 | ***0.002*** |
| Dissolved oxygen | 1 | 2242 | 4.5807 | ***0.001*** |
| Flushing time (days) | 1 | 2311 | 4.7219 | ***0.001*** |
| Catchment cleared (%) | 1 | 1752 | 3.5790 | ***0.001*** |
| Estuary surface area (km^2^) | 1 | 2568 | 5.2459 | ***0.001*** |
| Residuals | 67 | 32798 |  |  |
| **Fish hindgut** |  |  |  |  |
| Catchment area | 1 | 200669 | 7.8084 | ***0.002*** |
| pH | 1 | 67664 | 2.6329 | ***0.027*** |
| Urbanisation | 1 | 81573 | 3.1741 | ***0.023*** |
| Estuary volume | 1 | 72294 | 2.8131 | ***0.038*** |
| Residuals | 50 | 1284958 |  |  |
