# Supplementary material for: Spatial patterns in host-associated and free-living bacterial communities across six temperate estuaries
Source: FEMS Microbiol Ecol. 2023 Jun 2;99(7):fiad061. doi: 10.1093/femsec/fiad061 (PMC10284270; doi:10.1093/femsec/fiad061)
Supplement: fiad061_Supplemental_Files [file fiad061_supplemental_files.zip › Supp_data Figure Legends.docx]

Supplementary Figure Legends

Figure S1. Rarefaction curves for seawater, sediment and fish hindgut samples on raw sequencing data and after rarefaction to 4,276 reads.

Figure S2. Correlation heatmaps showing Spearman correlations between most abundant ASVs (top 50) from A) seawater, B) sediment and C) *Pelates sexlineatus* hindgut samples and environmental variables. Coloured circles indicate significant (p < 0.05) correlations, and blank spaces indicate no significant correlations. ASVs are identified to lowest possible taxonomic level. DO refers to dissolved oxygen and estuary SA to estuary surface area (km^2^). Created partially with BioRender.com.
